# Anesthesia Preferences and Outcomes in Saudi Arabian Healthcare: A Cross-Sectional Study

**DOI:** 10.7759/cureus.57340

**Published:** 2024-03-31

**Authors:** Sarah Rayyani, Hind Aljedani, Razan Kariri, Ghaida Alsuhim, Manal Madkhali, Hailah Oraybi, Shaima Assiri, Rand Alhayaza, Abdulqadir Madah

**Affiliations:** 1 College of Medicine, Jazan University, Jazan, SAU; 2 College of Medicine, King Abdulaziz University, Jeddah, SAU; 3 College of Medicine, King Khalid University, Abha, SAU; 4 Anesthesia, Dallah Hospital, Riyadh, SAU

**Keywords:** anesthesia training, saudi arabia, healthcare settings, elective surgeries, general anesthesia, regional anesthesia, anesthesia preferences

## Abstract

Background: Anesthesia choice is critical in ensuring optimal surgical outcomes and patient satisfaction. We aimed to investigate anesthesia preferences, trends, and outcomes in elective surgeries within Saudi Arabian healthcare settings.

Methods: A cross-sectional survey-based study was conducted among anesthesia residents and attending anesthesiologists across Saudi Arabia. Participants provided demographic information and responded to questions regarding anesthesia preferences, trends, and outcomes. Descriptive statistics were used to summarize the data, and logistic regression analysis was employed to identify factors associated with anesthesia preference.

Results: The survey was completed by 572 healthcare professionals in Saudi Arabia. Among participants, 51.7% (n=296) preferred general anesthesia, while 48.3% (n=276) favored regional anesthesia for elective surgeries. Factors influencing anesthesia choice included patient preference, surgical complexity, and resource availability. Over half of the respondents reported an increase in regional anesthesia preference over the past five years, although some perceived inadequate training in this area. Common barriers to regional anesthesia adoption included equipment availability, patient reluctance, and limited training opportunities. Postoperative recovery was perceived as quicker with regional anesthesia by 52.3% (n=299) of participants, with postoperative nausea and vomiting being the most common complication associated with general anesthesia. Multivariable logistic regression analysis revealed that participants above 50 years had lower odds of preferring regional anesthesia, while those perceiving training adequacy in regional anesthesia as adequate had higher odds of preferring it (OR=0.64, 95% CI: 0.41-0.98, p=0.041; OR=1.58, 95% CI: 1.21-2.05, p=0.001, respectively).

Conclusion: This study provides insights into anesthesia practice patterns in Saudi Arabian healthcare settings. Individualized anesthesia care, ongoing training in regional anesthesia, and evidence-based decision-making are essential to optimize perioperative outcomes and patient satisfaction.

## Introduction

Anesthesia plays a pivotal role in modern surgical practice, aiming to provide optimal patient comfort, safety, and surgical conditions. The choice between regional anesthesia and general anesthesia for elective surgeries remains a topic of ongoing debate, with considerations including patient preferences, surgical requirements, and perioperative outcomes [1]. 

Regional anesthesia, including techniques such as neuraxial blocks and peripheral nerve blocks, offers several advantages, including reduced systemic side effects, enhanced postoperative pain control, and potential for improved recovery and satisfaction [2-4]. Studies have demonstrated its efficacy and safety across various surgical procedures, particularly in orthopedic, gynecological, and urological surgeries [5,6]. However, regional anesthesia requires specialized skills and may not be suitable for all patients or surgical scenarios.

General anesthesia, characterized by unconsciousness and analgesia, remains the cornerstone of anesthesia practice for many surgical procedures. It provides complete control of the airway and enables the surgeon to perform complex interventions with minimal patient movement [7]. However, general anesthesia is associated with systemic effects such as postoperative nausea and vomiting, longer recovery times, and potential risks such as aspiration and adverse drug reactions [7].

In recent years, there has been a growing interest in regional anesthesia as an alternative or adjunct to general anesthesia, driven by advancements in regional anesthesia techniques, concerns about opioid-related complications, and emphasis on enhanced recovery after surgery protocols [8-11]. Training in regional anesthesia is essential to ensure safe and effective practice [12,13]. However, there may be variations in the quality and consistency of regional anesthesia training programs, both within and across countries [12,13]. Adequate training encompasses theoretical knowledge, practical skills, and clinical experience, supported by structured educational curricula and opportunities for hands-on training and mentorship.

While previous studies have examined the preferences, trends, and outcomes associated with regional anesthesia and general anesthesia in various healthcare settings [8-10], there is limited research specifically focusing on these aspects within the context of Saudi Arabian anesthesia practice. This study aims to investigate the preferences, trends, and outcomes associated with regional anesthesia versus general anesthesia in elective surgeries within Saudi Arabian healthcare settings.

## Materials and methods

Study design

A cross-sectional survey-based study was conducted to investigate the preferences, trends, and outcomes associated with regional anesthesia versus general anesthesia in elective surgeries within Saudi Arabian healthcare settings.

Study participants

A convenience sampling method was employed to recruit participants. Anesthesia residents and attending anesthesiologists practicing in various healthcare settings across Saudi Arabia were invited to participate in the survey. Invitations were sent via email and distributed through professional anesthesia networks and social media platforms. Participation was voluntary, and responses were anonymized to ensure confidentiality.

Survey instrument

The survey questionnaire was developed based on a comprehensive review of relevant literature, expert consultation, and pilot testing. The questionnaire comprised multiple-choice questions covering demographic information (e.g., age, gender, specialty, years of experience), anesthesia preferences and practices (e.g., preferred anesthesia type, frequency of using regional anesthesia), perceptions of trends and training in regional anesthesia, and perceptions of outcomes and complications associated with different anesthesia types.

Data collection procedure

Data were collected using an online survey platform. Participants were provided with a link to the survey and were asked to complete it at their convenience. Reminders were sent periodically to encourage participation. The survey remained open for a specified period, during which responses were collected.

Data analysis

Descriptive statistics were used to summarize the demographic characteristics of the participants, anesthesia preferences and practices, trends and training perceptions, and outcomes and complications. Logistic regression analysis was employed to explore associations between variables of interest and to identify factors influencing the preference for regional anesthesia over general anesthesia. Odds ratios (OR) with 95% confidence intervals (CI) were calculated to quantify the strength of associations.

## Results

Demographic characteristics of participants

A total of 572 participants completed the survey. The demographic characteristics of the participants are summarized in Table 1. The majority of respondents were aged between 31 and 40 years (n=238, 41.6%), with a slight predominance of male participants (n=308, 53.8%) over female participants (n=264, 46.2%). The most common specialty among participants was anesthesiology (n=412, 72.0%), followed by surgery (n=96, 16.8%). Regarding years of experience, the majority of respondents had between one and five years of experience (n=296, 51.7%).

**Table 1:** Demographic characteristics of participants (N=572). The table presents the distribution of demographic characteristics among participants who completed the survey. Values are reported as counts (n) and percentages (%). Percentages may not add up to 100% due to rounding.

Characteristic		Frequency (n)	Percentage (%)
Age	20–30 years	88	15.4
	31–40 years	238	41.6
	41–50 years	138	24.1
	Above 50 years	108	18.9
Gender	Male	308	53.8
	Female	264	46.2
Specialty	Anesthesiology	412	72.0
	Surgery	96	16.8
	Other	64	11.2
Years of experience	Less than 1 year	124	21.7
	1–5 years	296	51.7
	6–10 years	104	18.2
	More than 10 years	48	8.4

Anesthesia preferences and practices

The preferred type of anesthesia for elective surgeries varied among participants. Regional anesthesia was favored by 48.3% (n=276) of respondents, while 51.7% (n=296) preferred general anesthesia. Additionally, 35.7% (n=204) reported using regional anesthesia frequently, while 64.3% (n=368) used it occasionally. Factors influencing the choice between regional and general anesthesia included patient preference (29.5%, n=169), surgical complexity (41.2%, n=236), and resource availability (25.6%, n=147).

A majority of respondents (n=312) reported observing an increase in the preference for regional anesthesia over the past five years, while 22.9% (n=131) perceived the training and education in regional anesthesia within Saudi Arabian healthcare settings as inadequate. The most commonly reported barriers to the widespread adoption of regional anesthesia included limited availability of equipment (41.8%, n=239), patient reluctance (27.8%, n=159), and lack of training opportunities (35.2%, n=201) (Table 2).

**Table 2:** Trends in anesthesia preference over time and perceived training adequacy (N=572). The table illustrates the preferences and practices related to the use of regional anesthesia and general anesthesia among survey participants. Values are reported as frequencies (n) and percentages (%). Percentages may not add up to 100% due to rounding.

Variable		Frequency (n)	Percentage (%)
Preferred type	Regional anesthesia	276	48.3
	General anesthesia	296	51.7
Frequency of using regional anesthesia	Frequently	204	35.7
	Occasionally	368	64.3
Factors influencing choice between regional and general anesthesia	Patient preference	169	29.5
	Surgical complexity	236	41.2
	Anesthesiologist's preference	251	43.9
	Resource availability	147	25.6
	Complications of each type	72	12.4
Specific types of surgeries preferring regional anesthesia	Orthopedic surgeries	187	32.7
	Abdominal surgeries	290	50.7
	Thoracic surgeries	174	30.4
	Obstetric surgeries	405	70.8
Change in preference to regional anesthesia	Increase	312	54.5
	Decrease	100	17.5
Perceived training adequacy in regional anesthesia	Adequate	260	45.5
	Inadequate	312	54.5
Barriers to adoption of regional anesthesia	Lack of training	201	35.2
	Limited equipment	239	41.8
	Patient reluctance	159	27.8
	Surgeon's preference	140	24.5
	Time constraints	167	29.2

Outcomes and complications

Regarding postoperative recovery, 52.3% (n=299) of participants believed that regional anesthesia generally led to quicker recovery, while 25.2% (n=144) perceived no significant difference between regional and general anesthesia. The most commonly encountered complications associated with general anesthesia were postoperative nausea and vomiting (70.8%, n=405) (Table 3).

**Table 3:** Reported experience related to complications of anesthesia types (N=572). The table illustrates the previous experience encountered with complications and outcomes of regional anesthesia and general anesthesia among survey participants. Values are reported as frequencies (n) and percentages (%). Percentages may not add up to 100% due to rounding.

Variable		Frequency (n)	Percentage (%)
Type of anesthesia leading to quicker postoperative recovery	Regional anesthesia	299	52.3
	General anesthesia	129	22.5
	No significant difference	144	25.2
Encountered complications with regional anesthesia	Nerve injury	12	2.1
	Hematoma formation	98	17.1
	Infection	80	13.4
	Prolonged motor blockade	102	17.8
Encountered complications with general anesthesia	Nausea and vomiting	405	70.8
	Respiratory complications	385	67.3
	Hemodynamic instability	143	25.0
	Delayed emergence	88	15.4

Factors associated with anesthetic preference

We conducted a multivariable logistic regression analysis to explore the factors associated with the preference for regional anesthesia over general anesthesia. The results indicated that participants above 50 years of age had significantly lower odds of preferring regional anesthesia over general anesthesia compared to those aged 20-30 years (OR=0.64, 95% CI: 0.41-0.98, p=0.041). Additionally, participants who perceived the training adequacy in regional anesthesia as adequate had significantly higher odds of preferring regional anesthesia (OR=1.58, 95% CI: 1.21-2.05, p=0.001) (Table 3).

**Table 4:** Multivariable regression model of factors associated with preference for regional anesthesia. The table presents the results of multivariable logistic regression analysis identifying factors associated with the preference for regional anesthesia over general anesthesia among participants. Odds ratios with 95% confidence intervals are reported. Statistical significance is denoted by an asterisk for p-values < 0.05.

Variable		Odds ratio (95% Confidence interval)	P value
Age	20–30 years	Reference group	Not applicable
	31–40 years	0.85 (0.61-1.18)	0.327
	41–50 years	0.72 (0.49-1.05)	0.089
	Above 50 years	0.64 (0.41-0.98)	0.041*
Gender	Male	Reference group	Not applicable
	Female	1.15 (0.89-1.49)	0.289
Specialty	Anesthesiology	Reference group	Not applicable
	Surgery	0.78 (0.61-1.00)	0.052
	Other	0.92 (0.68-1.25)	0.614
Years of experience	Less than 1 year	Reference group	Not applicable
	1–5 years	1.20 (0.89-1.61)	0.228
	6–10 years	1.05 (0.71-1.55)	0.810
	More than 10 years	0.91 (0.54-1.54)	0.731
Perceived training adequacy	Adequate	1.58 (1.21-2.05)	0.001*
	Inadequate	0.92 (0.71-1.19)	0.535

## Discussion

The findings of this study provide valuable insights into the preferences, trends, and outcomes associated with regional anesthesia versus general anesthesia in elective surgeries within Saudi Arabian healthcare settings. The survey results revealed a balanced preference among anesthesia professionals for both regional anesthesia and general anesthesia, with approximately half of the respondents favoring each modality. This highlights the importance of individualized anesthesia care, tailored to patient needs, surgical requirements, and provider expertise. The relatively high frequency of using regional anesthesia, reported by over one-third of participants, reflects its growing acceptance and utilization in contemporary anesthesia practice.

Factors influencing the choice between regional anesthesia and general anesthesia included patient preference, surgical complexity, and resource availability. Patient-centered care, involving shared decision-making and informed consent, is essential to align anesthesia choice with patient preferences and values. Moreover, appropriate patient selection and thorough preoperative assessment are crucial to ensure the safety and efficacy of regional anesthesia techniques [14,15].

The majority of participants reported observing an increasing trend in the preference for regional anesthesia over the past five years. This trend may be attributed to several factors, including advancements in regional anesthesia techniques, evolving perioperative care paradigms such as enhanced recovery after surgery, and growing awareness of opioid-sparing analgesic strategies. However, the perception of training adequacy in regional anesthesia varied among participants, with approximately half considering the training to be inadequate. This underscores the need for standardized, competency-based training programs in regional anesthesia, encompassing both theoretical knowledge and practical skills [6-11].

Perceptions of postoperative recovery favored regional anesthesia, with over half of the participants believing it leads to quicker recovery compared to general anesthesia. This finding aligns with previous research demonstrating the benefits of regional anesthesia in terms of reduced postoperative pain, shorter hospital stays, and improved patient satisfaction [5-9]. However, it is essential to acknowledge the potential complications associated with regional anesthesia, including nerve injury, hematoma, and local anesthetic toxicity, albeit rare. Adequate patient monitoring, appropriate technique selection, and adherence to safety guidelines are essential to mitigate these risks [2,15].

Several limitations should be considered when interpreting the findings of this study. The cross-sectional design limits the ability to establish causality or temporal relationships between variables. Convenience sampling may introduce selection bias, potentially affecting the generalizability of the findings to the broader anesthesia community in Saudi Arabia. Moreover, reliance on self-reported data may be subject to recall bias and social desirability bias, influencing participant responses.

## Conclusions

In conclusion, this study sheds light on the nuanced landscape of anesthesia preferences, trends, and outcomes in elective surgeries within Saudi Arabian healthcare settings. Our findings underscore the importance of individualized anesthesia care, taking into account patient preferences, surgical requirements, and provider expertise. The observed trend towards increased utilization of regional anesthesia highlights the evolving nature of anesthesia practice and the potential benefits of opioid-sparing analgesic strategies. However, addressing the perceived inadequacies in regional anesthesia training and ensuring standardized practice guidelines are essential to maximize patient safety and optimize perioperative outcomes.
